# Response of Soil Bacterial Community Diversity and Composition to Time, Fertilization, and Plant Species in a Sub-Boreal Climate

**DOI:** 10.3389/fmicb.2020.01780

**Published:** 2020-08-05

**Authors:** Honghong Li, Petri Penttinen, Anu Mikkonen, Frederick L. Stoddard, Kristina Lindström

**Affiliations:** ^1^Ecosystems and Environment Research Programme, University of Helsinki, Helsinki, Finland; ^2^Helsinki Institute of Sustainability Science (HELSUS), University of Helsinki, Helsinki, Finland; ^3^Department of Microbiology, College of Resources, Sichuan Agricultural University, Chengdu, China; ^4^Kemira, Kemira Oyj, Espoo R&D Center, Espoo, Finland; ^5^Department of Agricultural Sciences and Viikki Plant Sciences Centre, Helsinki, Finland

**Keywords:** legume–grass mixture, bacterial community, seasonal change, pasture, sustainable agriculture

## Abstract

Pastures are an important part of crop and food systems in cold climates. Understanding how fertilization and plant species affect soil bacterial community diversity and composition is the key for understanding the role of soil bacteria in sustainable agriculture. To study the response of soil bacteria to different fertilization and cropping managements, a 3-year (2013–2015) field study was established. In the split-plot design, fertilizer treatment (unfertilized control, organic fertilizer, and synthetic fertilizer) was the main plot factor, and plant treatment [clear fallow, red clover (*Trifolium pratense*), timothy (*Phleum pratense*), and a mixture of red clover and timothy] was the sub-plot factor. Soil bacterial community diversity and composition, soil properties, and crop growth were investigated through two growing seasons in 2014 and 2015, with different nitrogen input levels. The community diversity measures (richness, Shannon diversity, and Shannon evenness) and composition changed over time (*P* < 0.05) and at different time scales. The community diversity was lower in 2014 than in 2015. The temporal differences were greater than the differences between treatments. The overall correlations of Shannon diversity to soil pH, NO${}_{3}^{-}$, NH${}_{4}^{+}$, and surplus nitrogen were positive and that of bacterial richness to crop dry matter yield was negative (*P* < 0.05). The major differences in diversity and community composition were found between fallow and planted treatments and between organic and synthetic fertilizer treatments. The differences between the planted plots were restricted to individual operational taxonomic units (OTUs). Soil moisture, total carbon content, and total nitrogen content correlated consistently with the community composition (*P* < 0.05). Compared to the unfertilized control, the nitrogen fertilizer loading enhanced the temporal change of community composition in pure timothy and in the mixture more than that in red clover, which further emphasizes the complexity of interactions between fertilization and cropping treatments on soil bacteria.

## Introduction

Grassy pastures are an important part of agricultural systems where ruminants are the main way for producing food under cold, dry, or other stressful conditions. Pastures for silage, hay, or grazing cover 26% of the arable region of Finland (Niemi and Väre, 2017), where the climate is defined as boreal and sub-boreal. To sustain or improve pasture production, farming systems have been widely intensified particularly through nitrogen (N) fertilizer inputs (Tilman et al., 2002; Stott and Gourley, 2016). However, reactive N as nitrate (NO${}_{3}^{-}$) in either synthetic fertilizer or manure is highly mobile and easily leached into waterways or denitrified, resulting in substantial environmental pollution (Philippot et al., 2007; Ju et al., 2009). Soil microbes can directly or indirectly affect plant productivity by regulating the acquisition of limiting nutrients and the farming impacts on the environment by moderating the biogeochemical processes (Verwaal et al., 2006; Van et al., 2008; Strickland et al., 2009). Soil bacteria are critical for N flows through biological nitrogen fixation (BNF) that provides N for crop growth, nitrification by oxidizing NH${}_{4}^{+}$ into NO${}_{3}^{-}$, denitrification by reducing NO${}_{3}^{-}$ into NO, N_2_O, and finally N_2_, and in dissimilatory NO${}_{3}^{-}$ reduction to ammonium (Philippot et al., 2007; Franche et al., 2009; Jia and Conrad, 2009; Yoshida et al., 2009; Robertson and Groffman, 2015).

Achieving or maintaining functional and diverse populations of soil organisms is one of the major criteria for sustainable land management (Dumanski et al., 1991). The effects of fertilizer and crop management on soil microbial communities may help us to understand the key roles of soil microbes on the biogeochemical processes of nutrient flows and crop production. Wang et al. (2017) reported a decrease in bacterial richness with synthetic fertilizer, and the simultaneous loss of soil biodiversity caused by intensification has raised concerns (Orgiazzi et al., 2016). In a comparison between grassland and managed fields, more intensive management resulted in greater differences in bacterial communities over time (Lauber et al., 2013). Generally, detecting the differences requires long-term studies (Shen et al., 2010; Hui et al., 2017). A meta-analysis by de Graaff et al. (2019) summarized that bacterial diversity was increased when synthetic N input rate was less than 150 kg ha^–1^ year^–1^, with organic N applied for more than 5 years, and the authors suggested that this was due to increased soil organic matter accumulation and retention. With elevated N input or across a N gradient, the community composition became progressively more distinct at higher N levels from those at no or lower levels of N fertilizer (Ramirez et al., 2010; Fierer et al., 2012). In addition, Soong et al. (2020) reported that soil organic carbon (C) content is the primary limitation for heterotrophic microbial communities.

The presence of N_2_-fixing legumes in legume–grass intercropping was considered as a component of sustainable intensification to enhance resource use efficiency and improve crop productivity (Tilman et al., 2002; Martin-Guay et al., 2018). Plant species could strongly affect the structure of rhizosphere microbial communities through root exudates (Haichar et al., 2008; Berg and Smalla, 2009). Nevertheless, little is known about the temporal response of soil bacterial community diversity and composition to the presence of legumes in field-grown crop mixtures with different fertilization treatments. Hence, we established a 3-year field study in the sub-boreal conditions of southern Finland. We have previously shown that the management and the season induced temporal changes in crop growth and soil properties, with the legume–grass mixture providing particular advantages over the pure stands of red clover (*Trifolium pratense*) and timothy (*Phleum pretense*) in terms of improved pasture yield and reduced potential N loss (Li et al., 2019). Given that the changes in soil organic C content, N availability, C/N ratio (Marschner et al., 2003; Ramirez et al., 2010), soil pH (Enwall et al., 2007; Shen et al., 2010), and soil moisture and temperature (Lauber et al., 2013) could drive the differences in microbial communities, we hypothesized that the response of community diversity measures (richness, Shannon diversity, and Shannon evenness) and composition would change over time and that the higher N input level in the third year would result in greater differences between treatments. Assuming that the advantages of the grass–legume mixture were linked to the differences in the biogeochemical processes of the nutrients caused by the bacterial community, we hypothesized that the soil bacterial communities in the mixture would be different from that in pure stands and that this would provide insight into the connections between soil bacteria and sustainable intensification of agriculture.

## Materials and Methods

### Field Establishment and Soil Sampling

The flat experimental field of 0.56 ha was at the Viikki Experimental Farm, University of Helsinki, Finland (60.227°N, 25.018°E, approximately 10 m above mean sea level). The soil texture was clay loam, with 32% clay, 36% silt, and 32% sand on average. The preceding crops were faba bean in 2010 with fertilizer N at 20 kg ha^–1^ year^–1^, followed by barley in 2011 and 2012 with fertilizer N at 60 kg ha^–1^ year^–1^. Prior to this experiment, soil pH was 6.41, total N content was 1.68 g kg ^–1^, total C content was 25.3 g kg^–1^, NO_3_–N content was 5.42 mg kg^–1^, and NH_4_–N content was 4.51 mg kg^–1^.

The experiment started in May 2013 and ended in September 2015. The split-plot design included 48 plots (four replicates × three fertilizer treatments × four crop treatments). Each replicate block of 18 m × 8 m was divided into 6 m × 8 m main plots for the fertilizer treatments: no fertilizer (control), organic (urine in 2014 and manure slurry in 2015), and synthetic [Ca(NO_3_)_2_]. The main plot was divided into four 6 m × 2 m sub-plots for the crop treatments: fallow, pure red clover, pure timothy, and a mixture of red clover and timothy. The weeds in the fallow were removed with a rotary tiller. Barley was grown in the surrounding field in all 3 years, with N fertilization at 80 kg ha^–1^ year^–1^. Fertilizer was applied manually twice per year. In 2014, with reduced nitrogen input, organic fertilizer was spread at 35 and 20 kg N ha^–1^ and synthetic fertilizer was spread at 40 and 20 kg N ha^–1^ on 7 May and 8 July, respectively. In 2015, with normal nitrogen input, both the organic and the synthetic fertilizers were spread at 75 and 75 kg N ha^–1^ on 1 June and 16 July, respectively. Except for the small difference at the first time point, the N inputs were consistent in the organic and the synthetic treatments. The surplus N (kg ha^–1^) was calculated as fertilization N input – crop N yield.

The surface soil (0–20 cm) was sampled twice annually in 2014 and 2015 as 16 subsamples per sub-plot and mixed into one composite sample. The sampling dates were June 2014, September 2014, July 2015, and September 2015. In total, 191 soil samples were investigated. Field-moist soil samples were passed through a 5 mm sieve and preserved at −20°C until DNA isolation and dried before the biophysiochemical analysis. Soil NH${}_{4}^{+}$ and NO${}_{3}^{-}$ were extracted with 2 M KCl and measured with Lachat QuickChem 8000 (Lachat Instruments, Milwaukee, WI, United States), soil pH and EC were determined in a 1:2.5 (w/w) soil–water mixture, and total C and N were determined with TruSpec Elemental Determinator (LECO, United States). The details were reported in Li et al. (2019).

### DNA Isolation and Amplicon Sequencing

Soil DNA was isolated from 0.25 g of soil with a Power Soil DNA Isolation Kit (MoBio, Carlsbad, CA, United States), following the manufacturer’s instructions. The quality of isolated DNA was checked with electrophoresis in 1% agarose gel, and the DNA was quantified fluorometrically using a PicoGreen dsDNA Quantification Reagent Kit (Molecular Probes, United States) in 96-well plates according to the manufacturer’s instructions. To amplify the V_3_ and V_4_ regions of the bacterial 16S rRNA gene, we used the primers 341F and 785R in duplicate 25 μl PCR reactions (Supplementary Table S1). After PCR, the amplicons from the duplicates were pooled and checked with electrophoresis in 1% agarose gel. The amplicons were sequenced using Illumina MiSeq sequencing (2 × 300 bp paired end) at the Institute of Biotechnology sequencing core facility, University of Helsinki.

### Bioinformatics

The sequences were processed using Mothur v.1.39.5 (Schloss et al., 2009) with standard operating procedures SOP (Kozich et al., 2013) in the Taito supercluster of the Finnish IT Centre for Science (CSC, Espoo, Finland). In total, 11,020,235 sequences were aligned against the SILVA reference database v.132. After removing the chimeras, 2,568,584 sequences were classified into operational taxonomic units (OTUs) at 97% similarity level against the SILVA database v.132, with a bootstrap value of 80% for taxonomic assignment. Finally, 27,535 OTUs were obtained after removing the singletons and subsampled into the sequencing depth of 4,680 sequences per sample. The number of OTUs, Shannon diversity, and Shannon evenness was calculated in Mothur (v.1.39.5) (Kozich et al., 2013).

### Statistical Analysis

All statistical analyses were performed in RStudio v. 1.1.383 (RStudio Team, 2016) with related packages based on R v. 3.5.0 (R Core Team, 2018). As the soil samples were collected from the 48 plots over four time points, the repeated-measures analysis of variance (rmANOVA) was performed to test the overall between-subjects and within-subject effects on soil bacterial community diversity and the relative abundances of individual taxa. Sampling time was treated as a repeated factor (a within-subject factor), block was a random factor, and fertilizer and crop treatments were fixed factors. As fertilizer was the main-plot factor, the overall main effect of fertilizer (*df* = 2) was tested against fertilizer × block (*df* = 6), and the effect of time × fertilizer was tested against time × fertilizer × block (*df* = 18). As crop was the sub-plot factor, the overall main effect of crop (*df* = 3) and the interaction of crop × fertilizer (*df* = 6) were tested against the main residual (*df* = 27). The effects of crop × time and crop × fertilizer × time were tested against the overall residual (*df* = 80).

To further test the fertilizer and the crop effects on soil bacterial community diversity and the relative abundance of individual taxon at each time point, the linear mixed-effects model was performed with *lme* function in *nlme* v.3.1-137 package (Pinheiro et al., 2018). In the model, according to the split-plot design, block was a random factor within block × fertilizer (frame), while fertilizer and crop factors were fixed factors with *lme* function (response variable ∼ fertilizer × crop, data = data, random = ∼ 1| block/frame). The normality of the distribution and the homogeneity of model residuals were tested by plotting the residuals against theoretical quantiles and fitted values. When the probability (*P*-value) of the effects was less than 0.05, the comparisons between two levels of the treatment and the Tukey tests were performed in package *lsmeans* (Lenth, 2016).

The repeated-measures correlations (rmcorr) were calculated to test the overall association of soil properties and crop growth characteristics with bacterial community diversity in package *rmcorr* v. 0.3.0 (Bakdash and Marusich, 2018). At each time point, the correlations of soil properties with bacterial community composition were tested by using *Mantel test* with the Spearman method.

Based on the Bray–Curtis dissimilarity between samples, a repeated-measures permutational multivariate analysis of variance (rm perMANOVA) was conducted using a permutation test with pseudo-*F* ratios (permutations = 999). This was performed using the *adonis* function in *vegan* v.2.5.2 (Oksanen et al., 2018) to test the community composition change over time and the overall treatment effects on the community composition. To know the community composition differences between treatments at each time point, perMANOVA was conducted at each time point. When *P* < 0.05, the comparisons between two levels of the treatment were performed with Pillai–Bartlett test (permutations = 999) in *RVAideMemoire* package (Hervé, 2018).

*DESeq2* package v.1.20.0 (Love et al., 2014) was used for detecting the differential OTUs (log_2_ fold change in relative abundance) between two levels of the treatment based on the not subsampled OTU abundances. The differential OTUs were selected by the adjusted *P* < 0.01. All figures were prepared using the packages *ggplot2* v.3.1.0 (Wickham, 2016) and *randomcoloR* v.1.1.0 (Ammar, 2017).

### Sequence Accession Numbers

The sequence files have been deposited in the European Nucleotide Archive with project accession number PRJEB32412.

## Results

**FIGURE 1:**
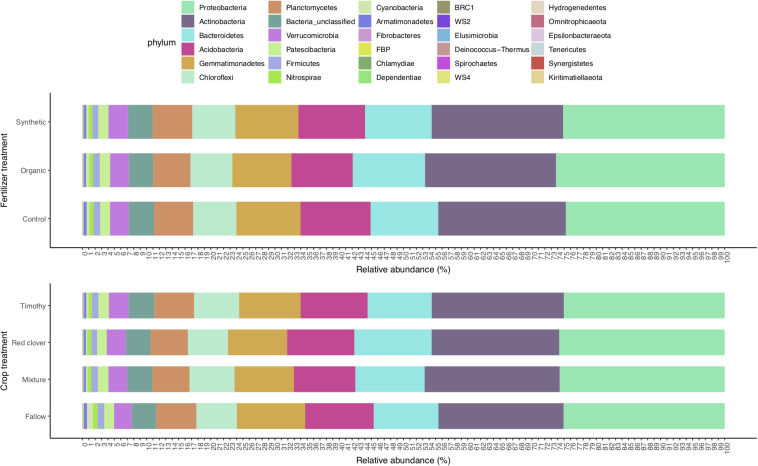
Soil bacterial phyla profile in a sub-boreal pasture. The abundances were defined based on 16S rRNA amplicon sequencing of soils from a split-plot field experiment with fertilizer and plant treatments. Fallow, no plant cover; mixture, red clover–timothy mixture; control, no fertilization; organic, organic fertilizer; synthetic, synthetic fertilizer.

The 27,535 16S rRNA gene OTUs were classified into 30 phyla (Figure 1), 82 classes (Supplementary Figure S1A), 198 orders (Supplementary Figure S1B), 355 families (Supplementary Figure S1C), and 698 genera (Supplementary Figure S1D). The most abundant phylum was *Proteobacteria* (relative abundance 0.25) that consisted of classes *Alphaproteobacteria* (0.163), *Gammaproteobacteria* (0.060), and *Deltaproteobacteria* (0.028). The other relatively abundant phyla were *Actinobacteria* (0.20), *Acidobacteria* (0.10), *Bacteroidetes* (0.11), *Gemmatimonadetes* (0.097), *Chloroflexi* (0.066), *Planctomycetes* (0.060), *Verrucomicrobia* (0.029), *Patescibacteria* (0.016), and *Firmicutes* (0.009) (Figure 1).

### Temporal Change of the Community Diversity and Composition

**FIGURE 2:**
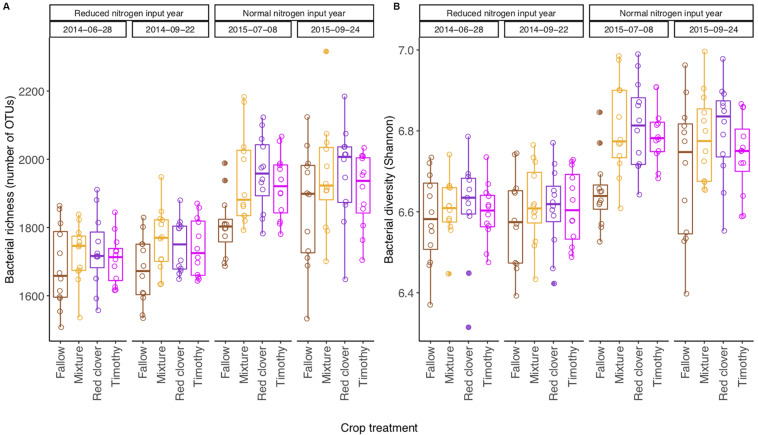
Boxplot (*n* = 12) of bacterial richness **(A)** and Shannon diversity **(B)** in a sub-boreal pasture over 2 years. The diversity indices were calculated based on 16S rRNA amplicon sequencing of soils from a split-plot field experiment with fertilizer (unfertilized control, organic fertilizer, and synthetic fertilizer) and plant (fallow, red clover, timothy, and a red clover–timothy mixture) treatments. Fallow: no plant cover; mixture, red clover–timothy mixture. The statistical analyses of differences between treatments at each time point and their temporal change are shown in Supplementary Table S2.

**FIGURE 3:**
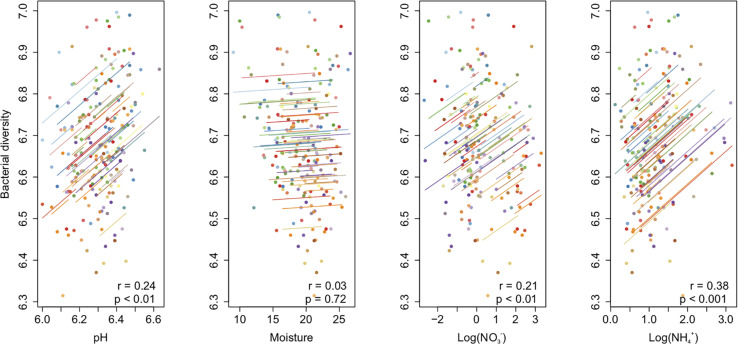
Repeated-measures correlation of soil bacterial diversity with soil pH, moisture, and NO_3_–N and NH_4_–N contents in a sub-boreal pasture. The diversity indices were calculated based on 16S rRNA amplicon sequencing of soils from a split-plot field experiment with fertilizer (unfertilized control, organic fertilizer, and synthetic fertilizer) and plant (fallow, red clover, timothy, and a red clover–timothy mixture) treatments.

The bacterial richness [*F*_(3,80)_ = 117.24, *P* < 0.001], Shannon diversity [*F*_(3,80)_ = 72.54, *P* < 0.001], and Shannon evenness [*F*_(3,80)_ = 27.53, *P* < 0.001] changed from year to year, but not from summer to autumn within a year (Table 1). The community diversity measures were generally higher in 2015 than in 2014 (Supplementary Table S2 and Figure 2). The Shannon diversity difference between crop treatments varied over time [*F*_(9,80)_ = 2.22, *P* < 0.05] (Table 1). For example, the Shannon diversity in fallow was 0.15 lower than in planted plots in July 2015 [*F*_(3,27)_ = 16.75, *P* < 0.001], whereas no difference was found in 2014 (Figure 2 and Supplementary Table S2). The overall repeated-measures correlation of the Shannon diversity with soil pH was 0.24 (*P* < 0.01), with NO${}_{3}^{-}$ it was 0.21 (*P* < 0.01), and with NH${}_{4}^{+}$ it was 0.38 (*P* < 0.001, Figure 3). The overall correlation of Shannon richness with crop dry matter yield was −0.20 (*P* < 0.05, Supplementary Figure S2) and with surplus nitrogen was 0.60 (*P* < 0.001, Supplementary Figure S3).

**TABLE 1:** Repeated-measures analysis of variance on soil bacterial community diversity in a sub-boreal pasture.

**Source**	**DF**	**denDF**	**Richness (number of operational taxonomic units)**		**Diversity (Shannon)**		**Evenness (Shannon)**	
			***F*-value**	***P*-value**	***F*-value**	***P*-value**	***F*-value**	***P*-value**
Fertilizer	2	6	1.18	0.37	1.85	0.24	3.02	0.12
Crop	3	27	18.13	< 0.001	12.00	< 0.001	5.31	0.005
Fert:crop	6	27	0.32	0.92	0.61	0.71	1.02	0.43
Time	3	80	117.24	< 0.001	72.54	< 0.001	27.53	< 0.001
Time:Fert	6	18	1.25	0.33	1.34	0.29	1.26	0.32
Time:crop	9	80	1.27	0.26	2.22	0.03	2.49	0.01
Time:Fert:crop	18	80	1.18	0.29	1.64	0.07	1.61	0.07

*The diversity indices were calculated based on 16S rRNA amplicon sequencing of soils from a split-plot field experiment with fertilizer (unfertilized control, organic fertilizer, and synthetic fertilizer) and plant (fallow, red clover, timothy, and a red clover–timothy mixture) treatments. Fert, fertilizer treatment; Time, sampling time point; DF, degree of freedom; denDF, DF of the denominator.*

**FIGURE 4:**
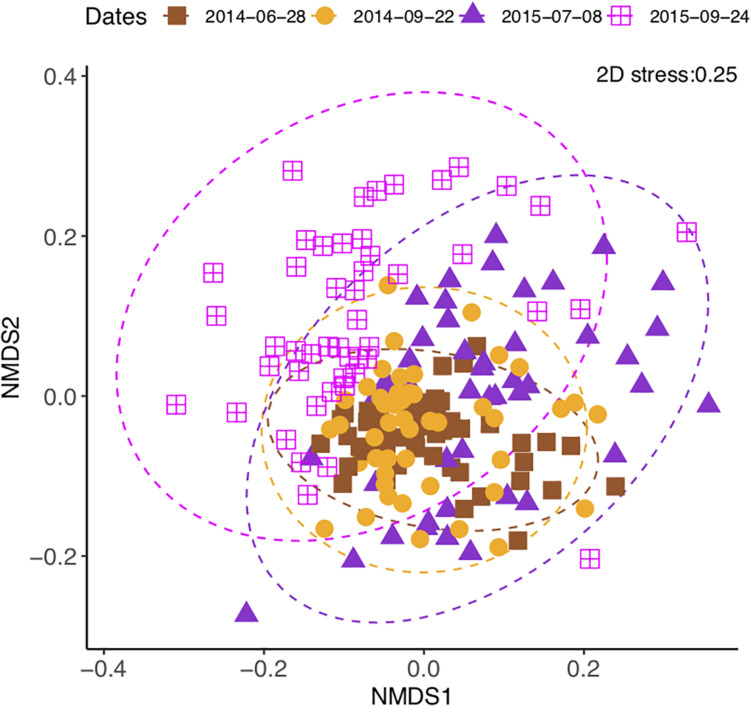
Non-metric multidimensional scaling of soil bacterial community change in a sub-boreal pasture over 2 years. The Bray–Curtis dissimilarities between samples were calculated based on 16S rRNA amplicon sequencing of soils from a split-plot field experiment with fertilizer (unfertilized control, organic fertilizer, and synthetic fertilizer) and plant (fallow, red clover, timothy, and a red clover–timothy mixture) treatments.

The community composition changed over time [perMANOVA, *F*_(3,80)_ = 7.42, *P* < 0.01], both year to year and from summer to autumn (Figure 4). The composition difference between crop treatments changed over time [perMANOVA, *F*_(9,80)_ = 1.27, *P* < 0.01]. The changes over time exceeded the differences between crop treatments (Supplementary Figure S4A) and fertilizer treatments (Supplementary Figure S4B). Soil moisture (*P* < 0.05 in all time points) and soil total N and total C (*P* < 0.01 at the measured time points) contents consistently correlated with the community composition (Table 2 and Supplementary Table S3).

**TABLE 2:** Mantel test (Spearman) of the correlation between soil physicochemical factors and bacterial community composition in a sub-boreal pasture.

**Environmental factors**	**June 2014**		**September 2014**		**July 2015**		**September 2015**	
	***r*_s_**	***P*-value**	***r*_s_**	***P*-value**	***r*_s_**	***P*-value**	***r*_s_**	***P*-value**
NO${}_{3}^{-}$	0.18	0.03	0.06	0.20	0.18	0.008	0.09	0.19
NH${}_{4}^{+}$	0.18	0.01	0.17	0.02	0.04	0.29	0.08	0.13
pH	0.24	0.001	0.21	0.006	0.06	0.11	0.09	0.08
EC	0.09	0.14	0.12	0.04	0.06	0.15	0.17	0.008
Moisture	0.27	0.001	0.13	0.01	0.14	0.02	0.25	0.001
TN	0.31	0.001	–	–	–	–	0.32	0.002
TC	0.28	0.001	–	–	–	–	0.30	0.001
C/N ratio	0.11	0.08	–	–	–	–	0.04	0.248

*The community compositions were defined based on 16S rRNA amplicon sequencing of soils from a split-plot field experiment with fertilizer (unfertilized control, organic fertilizer, and synthetic fertilizer) and plant (fallow, red clover, timothy, and a red clover–timothy mixture) treatments. TN, total nitrogen; TC, total carbon.*

**FIGURE 5:**
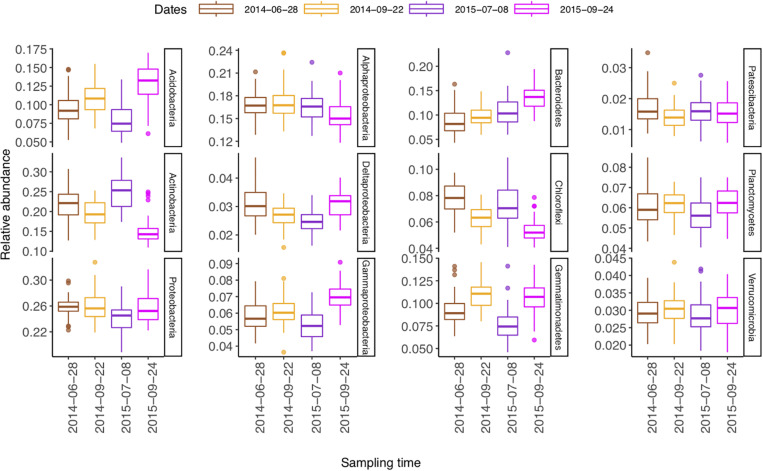
Boxplot (*n* = 48) of temporal change of the most abundant phyla (class) in a sub-boreal pasture. The abundances are defined based on 16S rRNA amplicon sequencing of soils from a split-plot field experiment with fertilizer and plant treatments. Fallow, no plant cover; mixture, red clover–timothy mixture; control, no fertilization; organic, organic fertilizer; synthetic, synthetic fertilizer. The statistical analyses of differences between treatments and changes over time are shown in Supplementary Table S4.

The relative abundances of *Actinobacteria* and *Chloroflexi* were higher in summer than in autumn, whereas those of *Acidobacteria*, *Gammaproteobacteria*, *Bacteroidetes*, *Gemmatimonadetes*, and *Planctomecetes* were lower (Figure 5).

### Differences Between Fertilizer Treatments

**FIGURE 6:**
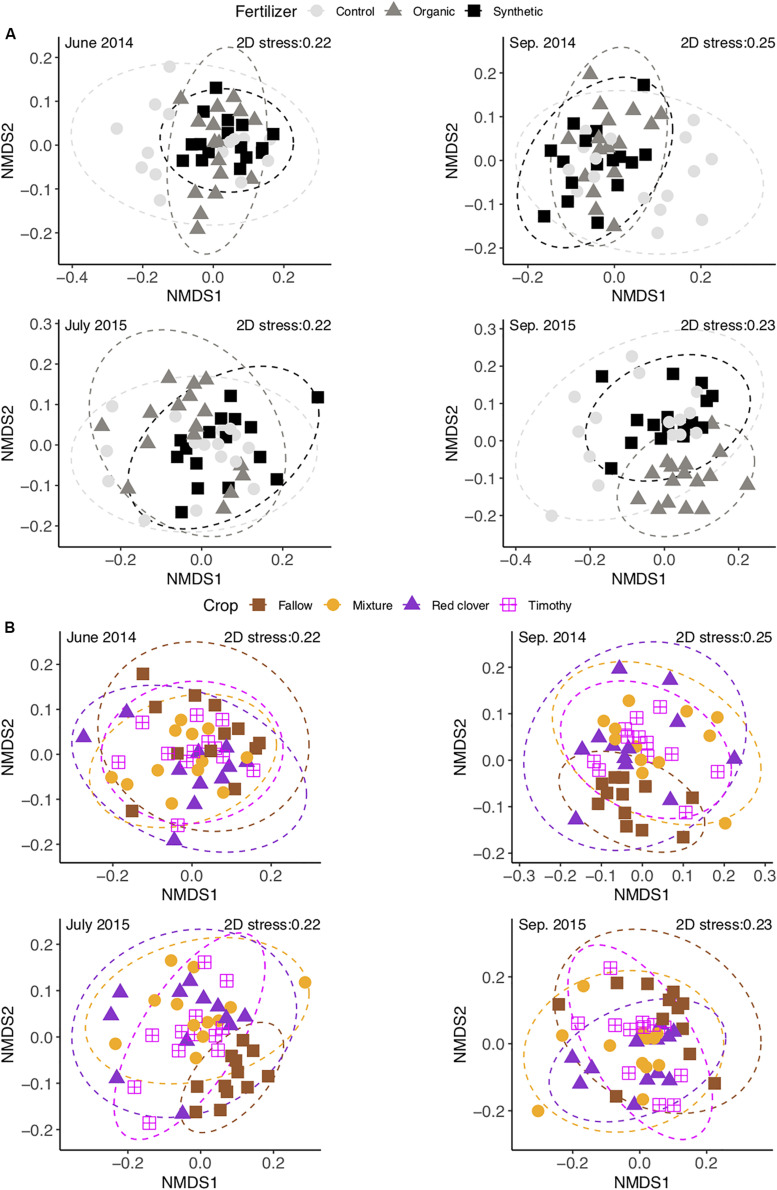
Non-metric multidimensional scaling of soil bacterial communities in a sub-boreal pasture under different fertilizer **(A)** and plant **(B)** treatments. The abundances are defined based on 16S rRNA amplicon sequencing of soils from a split-plot field experiment. Fallow, no plant cover; mixture, red clover–timothy mixture; control, no fertilization; organic, organic fertilizer; synthetic, synthetic fertilizer.

At the end of the experiment, the Shannon evenness in the organic fertilizer treatment was higher than in the control and the synthetic fertilizer treatments [*F*_(2,6)_ = 7.7, *P* < 0.05] (Supplementary Table S2). The community composition in the organic fertilizer treatment was different from those in the control and the synthetic fertilizer treatments [perMANOVA, *F*_(2,6)_ = 1.54, *P* < 0.01] (Figure 6A). With time, the number of differential OTUs in the synthetic vs. the organic fertilizer treatments increased gradually from 2 to 45 (Table 3), but no differential OTUs were found between the control and the synthetic fertilizer plots. With the exception of one *Chitinophagaceae* OTU, the relative abundances of *Bacteroidetes* OTUs were higher in the organic fertilizer treatment than in the synthetic fertilizer treatment (Supplementary Figure S5A). At the end of the experiment, the relative abundances of *Acidobacteria* and *Gemmatimonadetes* OTUs were higher in the synthetic fertilizer treatment than in the organic fertilizer treatment (Supplementary Figure S5A).

**TABLE 3:** Number of differential operational taxonomic units (OTUs) between treatments in a sub-boreal pasture.

**Between treatment**	**June 2014**	**September 2014**	**July 2015**	**September 2015**
Synthetic vs. organic	2	7	11	45
Control vs. organic	3	8	0	24
Control vs. synthetic	0	0	0	0
Mixture vs. fallow	18	51	65	10
Red clover vs. fallow	6	27	41	17
Timothy vs. fallow	16	64	50	35
Red clover vs. timothy	5	1	0	3
Red clover vs. mixture	0	0	0	0
Timothy vs. mixture	3	0	0	0

*The OTUs were defined based on 16S rRNA amplicon sequencing of soils from a split-plot-design field experiment with fertilizer (unfertilized control, organic fertilizer, and synthetic fertilizer) and plant (fallow, red clover, timothy, and a red clover–timothy mixture) treatments.*

The relative abundances of *Acidobacteria* were generally higher in the control and the synthetic fertilizer treatments than in the organic fertilizer treatment (Supplementary Table S4). At the end of the experiment, the relative abundances of *Proteobacteria* and *Fibrobacteres* were higher in the organic fertilizer treatment than in the synthetic fertilizer treatment (*P* < 0.05), whereas those of *Acidobacteria* and *Chloroflexi* were higher in the control treatment than in the organic fertilizer treatment (*P* < 0.05) (Supplementary Table S4).

### Differences Between Crop Treatments

In 2015, the community diversity measures in fallow plots were lower than those in planted plots (*P* < 0.05) (Supplementary Table S2). At each time point, the community composition in the fallow treatment was different from those in the planted treatments [perMANOVA, *F*_(3,26)_ = 1.53 in June 2014, *F*_(3,27)_ = 1.65 in September 2014, *F*_(3,27)_ = 1.83 in July 2015, and *F*_(3,27)_ = 1.64 in September 2015; *P* < 0.01] (Figure 6B and Supplementary Figure S6). The relative abundance of *Fibrobacteres* was higher and that of *Nitrospirae* was lower in the mixture and timothy treatments than in the fallow and red clover treatments (*P* < 0.05) (Figure 1 and Supplementary Table S4). The number of differential OTUs between the crop treatments changed over time (Table 3). At the end of the experiment, the number of differential OTUs was highest in timothy vs. fallow and lowest in red clover vs. timothy (Table 3). The differential OTUs in red clover vs. timothy treatments were mainly from the orders *Flavobacteriales*, *Rhizobiales*, and *Saccharimonadales*. Among the differential OTUs, the relative abundances of *Gemmatimonadetes* OTUs were higher in the fallow treatment than in the planted treatments (Supplementary Figure S5B). Compared to the unfertilized control, fertilization enhanced the temporal change in community composition in the mixture and the timothy plots (Supplementary Figure S7).

## Discussion

We studied the dynamic response of soil bacteria to three fertilization and four crop management practices over four time points in a 3-year field study. As expected, the community diversity and the composition changed over time. The community diversity and the composition changed at different time scales, with the diversity changing between years, whereas the composition also changed between seasons within a year. Similarly, Hillebrand et al. (2018) reported that changes in species richness were often decoupled from the changes in composition.

The temporal change of the communities exceeded the differences between the fertilizer and the crop treatments, as also found in a very different climate in California, United States (Cruz-Martínez et al., 2009). Diversity increased slightly in all the treatments and correlated positively with soil NO${}_{3}^{-}$ and NH${}_{4}^{+}$ contents. The opposite and the negative correlations were found when the study was based on a single time point (Zeng et al., 2016), which emphasizes the importance of knowing the overall driving factors of diversity through time series analyses (Lauber et al., 2013). Microbial diversity is not a quantitative measurement with absolutes that require caution when comparing directly across different methods or studies (Mikkonen, 2012; Shade, 2017). Nevertheless, in accordance with our hypothesis, the higher N input in the third year resulted in greater differences between treatments: the number of OTUs differentiating organic from synthetic fertilizer treatments increased gradually from two in June 2014 to 45 in September 2015. The quality and the quantity of organic C influence the soil microbiome (Fierer, 2017), which may explain the OTU level differences between organic fertilization and other treatments. In contrast, no OTUs separated the control from the synthetic fertilizer treatment. The soil bacterial communities are primarily organic C limited, and only secondarily N limited (Soong et al., 2020), which may explain the similarity of the communities in the control and the synthetic fertilizer treatment. According to the report from Fierer et al. (2007), the continuous C input in the organic fertilizer treatment may have contributed to the higher abundances of the copiotrophic *Bacteroidetes* OTUs. The communities in the organic fertilizer plots were more even than those in the control and the synthetic fertilizer plots, as also found by Sun et al. (2004).

The community diversity and the composition differences were mainly between fallow and planted treatments. The differences between planted treatments were restricted to individual OTUs. The relative abundances of differential *Flavobacteriales* and *Rhizobiales* OTUs were higher in the red clover treatment than in the timothy treatment. Even though differences in absolute abundances cannot be concluded from differences in relative abundances (Gloor et al., 2017), it is likely that *Rhizobiales* abundances could have increased due to their capability to live in symbiosis with legumes (Lindström et al., 2019).

We found that the soil total C, total N, and moisture contents consistently correlated with the community differences between treatments. The differences between fallow and planted plots could be attributed to the higher soil C content under canopies and differences in soil moisture and temperature linked to crop shading (Trivedi et al., 2019). Soil moisture can directly affect microbial activity, decomposition rates, and nutrient diffusion rates (Manzoni et al., 2012). The higher relative abundance of *Gemmatimonadetes* in fallow may have been due to lower moisture since this phylum prefers drier soil (Fawaz, 2013). Other composition differences included the higher relative abundances of *Planctomycetes* and the lower relative abundances of *Chloroflexi* and *Verrucomicrobia* in the fallow than in the planted treatments. The differences in composition may indicate differences in the rates of biogeochemical processes (Schimel, 2001). However, a deeper understanding of the parallel changes between bacterial community compositions and the process rates requires further studies.

Generally, the soil had lower NO_3_–N content and higher moisture content in the autumn than in the summer (Li et al., 2019). From summer to autumn, the relative abundances of *Acidobacteria* and *Bacteroidetes* increased, while that of *Actinobacteria* decreased. In contrast, Lauber et al. (2013) found an opposite pattern across land-use types, indicating that the interaction between the local climate and the agricultural managements on soil microbiome is complex. In the present experiment, fertilization enhanced the temporal changes in community composition in the mixture and the timothy plots but not in the red clover plots, which further emphasizes the complexity of interactions among fertilizer and cropping managements in affecting soil bacterial communities.

## Conclusion

In conclusion, we investigated the responses of soil bacterial diversity and community composition to pasture fertilizer and crop management procedures. Increased nitrogen input in the third year promoted diversity, which was associated with the overall increased pH as well as the increased availability of mineral N. Major differences in diversity and community composition were found between fallow and planted treatments, but not among the planted treatments, in spite of the higher botanical biodiversity in the mixture plots over the pure-stand plots. The relative abundances of individual OTUs differed between planted treatments and between organic and synthetic fertilizer treatments, such as the higher relative abundance of *Rhizobiales* OTUs in the clover-containing plots than in the timothy plots and the higher relative abundance of copiotrophic *Bacteroidetes* OTUs in the organic fertilizer plots than in the synthetic fertilizer plots. This suggests that the relative abundances of individual OTUs, in contrast to overall community diversity and composition, may help us to understand the mechanisms of bacterial response to agricultural practices and the possible connections of the response to the sustainability of agricultural practices.

## Data Availability Statement

The datasets generated for this study can be found in the European Nucleotide Archive (ENA) with accession number PRJEB32412.

## Author Contributions

HL did the soil DNA isolation, PCR, and sequences analysis under the supervision of AM and PP. HL did the statistical analysis and drafted the manuscript. All the authors agreed with the final version of the manuscript, contributed to interpreting the results, and revised the manuscript critically.

## Conflict of Interest

AM was employed by Kemira.

The remaining authors declare that the research was conducted in the absence of any commercial or financial relationships that could be construed as a potential conflict of interest.
