# Supplementary material for: Response of Soil Bacterial Community Diversity and Composition to Time, Fertilization, and Plant Species in a Sub-Boreal Climate
Source: Front Microbiol. 2020 Aug 5;11:1780. doi: 10.3389/fmicb.2020.01780 (PMC7419661; doi:10.3389/fmicb.2020.01780)
Supplement: Supplementary file 1 [file Table_1.pdf]

Response of soil bacterial community diversity and composition to time, fertilization and plant species in a sub-boreal climate

Honghong Li[1,2*], Petri Penttinen[1,3*], Anu Mikkonen[4], Frederick L. Stoddard[2,5], Kristina Lindström[1, 2]

1 Ecosystems and Environment Research Programme, University of Helsinki, PO Box 65, FI-00014 Helsinki, Finland
2 Helsinki Institute of Sustainability Science (HELSUS), University of Helsinki, Finland
3 Department of Microbiology, College of Resources, Sichuan Agricultural University, Chengdu 611130, China
4 Kemira, Kemira Oyj, Espoo R&D Center, Luoteisrinne 2, 00271 Espoo, Finland
5 Department of Agricultural Sciences and Viikki Plant Science Centre, PO Box 27, FI-00014 Helsinki, Finland

Contents:

Tables:

Table S1 Information of primers and PCR program

Table S2 Analyses of variance (ANOVA) (mean ± SE) of soil bacterial community diversity measures at each time point in soils from a split-plot field experiment with fertilizer (unfertilized control, organic fertilizer and synthetic fertilizer) and plant (fallow, red clover, timothy and a red clover – timothy mixture) treatments.

Table S3 Mantel test of the correlation (Spearman) between soil physicochemical factors and bacterial community composition.

Table S4 Analyses of variance (ANOVA) (mean ± SE) of relative abundances of most abundant taxa (class, order) at each time point in soils from a split-plot field experiment with fertilizer (unfertilized control, organic fertilizer and synthetic fertilizer) and plant (fallow, red clover, timothy and a red clover – timothy mixture) treatments.

Figures:

Figure S1 Soil bacterial taxonomy information on Class (A), Order (B), Family (C), and Genus (D) level.

Figure S2 Overall repeated measures correlation of bacterial community diversity measures to crop dry matter yield.

Figure S3 Dynamic change of surplus nitrogen (kg N ha^{-1}) (Fertilization N – Crop N yield) under

the treatment (A) and overall repeated measures correlations of surplus nitrogen (Fertilization N – Crop N yield) to bacterial community diversity measures (B).

Figure S4 Nonmetric multidimensional scaling (NMDS) of soil bacterial community composition temporal change within fertilizer (A) and crop (B) treatment. Fallow: no plant cover; mixture, red clover – timothy mixture; Control: no fertilization; organic: organic fertilizer; synthetic: synthetic fertilizer.

Figure S5 Log_2fold change of differential OTUs in synthetic vs. organic fertilizer treatment over time (A), and planted plots vs. fallow and red clover vs. timothy at the end of the experiment (B). Each coloured circle represents an OTU. Control: no fertilization; organic: organic fertilizer; synthetic: synthetic fertilizer. Fallow: no plant cover; mixture, red clover – timothy co-cultivation.

Figure S6 Nonmetric multidimensional scaling (NMDS) of soil bacterial community composition under different plant treatments in terms of NMDS2 to NMDS3 in June 2014 and Sept. 2015. Fallow: no plant cover; mixture, red clover – timothy mixture;

Figure S7 Temporal changes in Bray-Curtis dissimilarity in plant treatments within fertilizer treatments. The changes were calculated based on the averaged pairwise distance from June 2014 in percent difference. Fallow: no plant cover; mixture, red clover – timothy co-cultivation; Control: no fertilization; organic: organic fertilizer; synthetic: synthetic fertilizer.

Tables

Table S1 Information of primers and PCR program

Primers	Information
Illum_341F_1	ATCTACACTCTTTCCCTACACGACGCTCTTCCGATCTCCTACGGGNGGCWGCAG
Illum_341F_2	ATCTACACTCTTTCCCTACACGACGCTCTTCCGATCTgtCCTACGGGNGGCWGCAG
Illum_341F_3	ATCTACACTCTTTCCCTACACGACGCTCTTCCGATCTagagCCTACGGGNGGCWGCAG
Illum_341F_4	ATCTACACTCTTTCCCTACACGACGCTCTTCCGATCTtagtgtCCTACGGGNGGCWGCAG
Illum_785R_1	GTGACTGGAGTTCAGACGTGTGCTCTTCCGATCTGACTACHVGGGTATCTAATCC
Illum_785R_2	GTGACTGGAGTTCAGACGTGTGCTCTTCCGATCTaGACTACHVGGGTATCTAATCC
Illum_785R_3	GTGACTGGAGTTCAGACGTGTGCTCTTCCGATCTtctGACTACHVGGGTATCTAATCC
Illum_785R_4	GTGACTGGAGTTCAGACGTGTGCTCTTCCGATCTctgagtgGACTACHVGGGTATCTAATCC

PCR program

1st PCR reaction	1 reaction (µl)	PCR program		
5 x Buffer	5	1	98 °C	30 s
dNTPs (10 mM)	0.5	2	98 °C	10 s
pA_Illum_341F 4 mix (10 µM)	0.5	3	65 °C	30 s
pD'_Illum_785R 4 mix (10µM)	0.5	4	72 °C	10 s
polym.2 U/µL	0.25	5	go to 2	17 times
MQ	17.25	6	72 °C	5 min
Template DNA	1	7	4 °C	∞
Total	25			

Table S2 Analyses of variance (ANOVA) (mean ± SE) of soil bacterial community diversity measures at each time point in soils from a split-plot field experiment with fertilizer (unfertilized control, organic fertilizer and synthetic fertilizer) and plant (fallow, red clover, timothy and a red clover – timothy mixture) treatments.

Treatment		Richness				Shannon diversity				Shannon evenness			
		2014		2015		2014		2015		2014		2015	
		June	Sept.	July	Sept.	June	Sept.	July	Sept.	June	Sept.	July	Sept.
Control		1534	1549	1601	1598	6.52	6.52	6.61	6.57	0.889	0.889	0.896	0.891b
Organic		1545	1545	1655	1669	6.53	6.53	6.65	6.68	0.889	0.889	0.898	0.900a
Synthetic		1502	1497	1575	1576	6.50	6.47	6.58	6.55	0.888	0.885	0.894	0.890b
SEM		71	72	85	86	0.09	0.09	0.11	0.09	0.007	0.008	0.009	0.006
Fallow		1510	1485b	1526b	1570b	6.50	6.52	6.50b	6.55b	0.889	0.889	0.887b	0.891b
Mixture		1535	1551a	1633a	1639a	6.52	6.52	6.65a	6.62a	0.889	0.887	0.899a	0.895ab
Red clover		1536	1539ab	1646a	1641a	6.52	6.52	6.65a	6.64a	0.889	0.887	0.899a	0.897a
Timothy		1529	1546ab	1636a	1607ab	6.52	6.49	6.65a	6.59ab	0.889	0.888	0.899a	0.892ab
SEM		74	72	83	100	0.09	0.10	0.09	0.11	0.007	0.009	0.008	0.007
Tests of between-subject effects													
Treatment	DF	Significance level											
Fertilizer	2	ns	ns	ns	ns	ns	ns	ns	ns	ns	ns	ns	*
Crop	3	ns	*	***	**	ns	ns	***	**	ns	ns	***	*
Fert × Crop	6	ns	ns	**	ns	ns	ns	**	*	ns	ns	*	*

Sept., September; SEM, standard error of means; Fert, fertilizer treatment; DF, degrees of freedom; ns, not significant; * when $P < 0.05$, ** when $P < 0.01$, *** when $P < 0.001$; Different letters in a column indicate significant differences between fertilizer and crop treatment. The denominator DF of fertilizer, crop, and Fert × Crop are 6, 27, and 27 respectively. Since there was one missing value in June 2014, the denominator DF were 6, 26, and 26 respectively.

Table S3 Mantel test of the correlation (Spearman) between soil physicochemical factors and bacterial community composition.

Environmental factors	Control r_s	Control *P*-value	Organic r_s	Organic *P*-value	Synthetic r_s	Synthetic *P*-value
NO_3^-	-0.08	0.89	0.23	0.001	0.18	0.01
NH_4^+	0.10	0.04	0.04	0.23	0.24	0.003
pH	0.09	0.05	0.08	0.06	0.05	0.16
EC	0.10	0.02	0.05	0.12	0.20	0.002
Moisture	0.29	0.001	0.24	0.001	0.12	0.01

Environmental factors	Fallow r_s	Fallow *P*-value	Mixture r_s	Mixture *P*-value	Red clover r_s	Red clover *P*-value	Timothy r_s	Timothy *P*-value
NO_3^-	0.09	0.10	0.20	0.01	0.05	0.27	-0.005	0.51
NH_4^+	0.08	0.13	0.06	0.20	0.04	0.30	0.18	0.002
pH	0.15	0.01	0.13	0.02	0.14	0.006	0.09	0.08
EC	0.12	0.01	0.14	0.02	0.15	0.02	0.15	0.006
Moisture	0.13	0.01	0.25	0.001	0.28	0.001	0.24	0.001

Table S4 Analyses of variance (ANOVA) (mean ± SE) of relative abundances of most abundant taxa (class, order) at each time point in soils from a split-plot field experiment with fertilizer (unfertilized control, organic fertilizer and synthetic fertilizer) and plant (fallow, red clover, timothy and a red clover – timothy mixture) treatments.

Treatment		*Proteobacteria*				*Actinobacteria*				*Bacteroidetes*			
		2014		2015		2014		2015		2014		2015	
		June	Sept.	July	Sept.	June	Sept.	July	Sept.	June	Sept.	July	Sept.
Control		0.25	0.26	0.24	0.25b	0.217	0.186	0.241	0.143	0.09	0.10	0.10	0.13
Organic		0.26	0.26	0.25	0.27a	0.222	0.193	0.246	0.144	0.08	0.10	0.11	0.15
Synthetic		0.26	0.25	0.24	0.25b	0.212	0.208	0.251	0.156	0.09	0.09	0.11	0.13
SEM		0.01	0.02	0.02	0.02	0.02	0.01	0.01	0.006	0.01	0.005	0.01	0.005
Fallow		0.26	0.25	0.24	0.25	0.188b	0.207a	0.246	0.135	0.09a	0.09	0.08c	0.139ab
Mixture		0.26	0.27	0.24	0.26	0.233a	0.188b	0.257	0.168	0.07b	0.10a	0.11ab	0.128b
Red clover		0.26	0.27	0.24	0.26	0.222a	0.192ab	0.223	0.150	0.10a	0.10a	0.13a	0.145a
Timothy		0.26	0.25	0.24	0.25	0.226a	0.195ab	0.258	0.138	0.08ab	0.09a	0.10bc	0.134ab
SEM		0.01	0.02	0.02	0.02	0.02	0.01	0.01	0.006	0.01	0.005	0.01	0.005
Tests of Between-Subjects effects													
Treatment	DF	Significance level											
Fertilizer	2	ns	ns	ns	*	ns	ns	ns	ns	ns	ns	ns	ns
Crop	3	ns	ns	ns	ns	**	**	ns	ns	**	ns	***	*
Fert × Crop	6	ns	ns	ns	ns	ns	**	*	ns	ns	ns	**	**
Tests of Within-Subjects effects													
Treatment	DF	Significance level											
Time	3			***				***				***	

Treatment		*Acidobacteria*				*Gemmatimonadetes*				*Chloroflexi*			
		2014		2015		2014		2015		2014		2015	
		June	Sept.	July	Sept.	June	Sept.	July	Sept.	June	Sept.	July	Sept.
Control		0.10	0.11	0.08	0.14a	0.09	0.11	0.08	0.11	0.08	0.06	0.07	0.054a
Organic		0.09	0.10	0.07	0.11b	0.09	0.11	0.07	0.10	0.08	0.06	0.07	0.048b
Synthetic		0.10	0.11	0.08	0.13ab	0.09	0.11	0.08	0.11	0.08	0.06	0.07	0.051ab
SEM		0.006	0.008	0.006	0.008	0.005	0.006	0.005	0.004	0.006	0.002	0.006	0.002
Fallow		0.10a	0.10	0.09	0.13	0.10a	0.11	0.090a	0.12a	0.077ab	0.058b	0.066b	0.048b
Mixture		0.08b	0.11	0.07	0.12	0.09ab	0.11	0.067b	0.10b	0.083a	0.064ab	0.076ab	0.055a
Red clover		0.09ab	0.11	0.08	0.13	0.08b	0.10	0.074ab	0.10b	0.072b	0.059b	0.069ab	0.048b
Timothy		0.09ab	0.11	0.07	0.13	0.08b	0.11	0.075ab	0.11ab	0.076ab	0.069a	0.078a	0.052ab
SEM		0.006	0.008	0.006	0.008	0.005	0.006	0.005	0.004	0.006	0.002	0.006	0.002
Tests of Between-Subjects effects													
Treatment	DF	Significance level											
Fertilizer	2	ns	ns	ns	*	ns	ns	ns	ns	ns	ns	ns	*
Crop	3	*	ns	ns	ns	**	ns	*	**	*	**	*	**
Fert × Crop	6	ns	ns	ns	ns	ns	ns	*	ns	ns	ns	ns	**
Tests of Within-Subjects effects													
Treatment	DF	Significance level											
Time	3			***				***				***	

Treatment	DF	*Planctomycetes*				*Verrucomicrobia*				*Patescibacteria*			
		2014		2015		2014		2015		2014		2015	
		June	Sept.	July	Sept.	June	Sept.	July	Sept.	June	Sept.	July	Sept.
Control		0.059	0.062	0.058	0.065	0.027	0.030	0.028	0.030	0.017	0.013	0.015	0.016
Organic		0.060	0.059	0.053	0.060	0.030	0.030	0.028	0.029	0.017	0.016	0.018	0.014
Synthetic		0.062	0.064	0.057	0.064	0.031	0.030	0.029	0.030	0.017	0.012	0.017	0.016
SEM		0.003	0.002	0.002	0.003	0.005	0.005	0.005	0.005	0.004	0.004	0.004	0.004
Fallow		0.061	0.065	0.060a	0.062	0.029	0.028b	0.025b	0.027b	0.017	0.014	0.013b	0.016
Mixture		0.058	0.060	0.052b	0.064	0.031	0.030ab	0.028ab	0.030ab	0.019	0.014	0.017ab	0.015
Red clover		0.057	0.061	0.057ab	0.061	0.028	0.031ab	0.031a	0.029ab	0.016	0.013	0.017ab	0.014
Timothy		0.065	0.062	0.055ab	0.065	0.030	0.032a	0.029ab	0.033a	0.016	0.014	0.019a	0.015
SEM		0.003	0.002	0.002	0.003	0.005	0.005	0.005	0.005	0.004	0.004	0.004	0.004
Tests of Between-Subjects effects													
Treatment	DF	Significance level											
Fertilizer	2	ns	ns	ns	ns	ns	ns	ns	ns	ns	ns	ns	ns
Crop	3	ns	ns	*	ns	ns	*	*	*	ns	ns	*	ns
Fert × Crop	6	ns	ns	ns	ns	ns	ns	ns	ns	ns	ns	ns	ns
Tests of Within-Subjects effects													
Treatment	DF	Significance level											
Time	3	***				ns				*			

Treatment	DF	*Bacteria_unclassified*				*Cyanobacteria*				*Fibrobacteres*			
		2014		2015		2014		2015		2014		2015	
		June	Sept.	July	Sept.	June	Sept.	July	Sept.	June	Sept.	July	Sept.
Control		0.04	0.04	0.04	0.038	0.002	0.003	0.005	0.005	0.0005	0.0007	0.0007	0.0005b
Organic		0.04	0.04	0.04	0.035	0.002	0.002	0.013	0.002	0.0004	0.0005	0.0007	0.0014a
Synthetic		0.04	0.04	0.04	0.037	0.002	0.002	0.006	0.002	0.0008	0.0005	0.0009	0.0008b
SEM		0.001	0.001	0.001	0.001	0.0003	0.0004	0.003	0.0002	0.0002	0.0001	0.0002	0.0001
Fallow		0.042a	0.037ab	0.037ab	0.035	0.003	0.005a	0.023a	0.006a	0.0002c	0.0002b	0.0004c	0.0004b
Mixture		0.041ab	0.036ab	0.036ab	0.038	0.002	0.001b	0.002b	0.002ab	0.0006ab	0.0009a	0.0008ab	0.0014a
Red clover		0.040ab	0.035b	0.035b	0.035	0.002	0.002b	0.002b	0.001b	0.0003bc	0.0003b	0.0005bc	0.0007b
Timothy		0.038b	0.039a	0.039a	0.039	0.002	0.003ab	0.005a	0.002ab	0.0011a	0.0008a	0.0010a	0.0010a
SEM		0.001	0.001	0.001	0.001	0.0003	0.0004	0.002	0.0003	0.0002	0.0001	0.0002	0.0001
Tests of Between-Subjects effects													
Treatment	DF	Significance level											
Fertilizer	2	ns	ns	ns	ns	ns	ns	ns	ns	ns	ns	ns	**
Crop	3	*	*	*	ns	ns	**	*	*	**	***	***	***
Fert × Crop	6	ns	**	**	ns	ns	ns	ns	ns	ns	ns	ns	ns
Tests of Within-Subjects effects													
Treatment	DF	Significance level											
Time	3	***				ns				***			

Treatment	DF	*Alphaproteobacteria*				*Gammaproteobacteria*				*Deltaproteobacteria*			
		2014		2015		2014		2015		2014		2015	
		June	Sept.	July	Sept.	June	Sept.	July	Sept.	June	Sept.	July	Sept.
Control		0.167	0.162	0.160	0.142	0.059	0.062	0.052	0.071ab	0.030	0.026	0.024ab	0.033
Organic		0.176	0.171	0.168	0.166	0.058	0.062	0.055	0.072a	0.032	0.028	0.027a	0.030
Synthetic		0.162	0.176	0.167	0.152	0.058	0.059	0.050	0.068b	0.031	0.026	0.021b	0.030
SEM		0.004	0.006	0.005	0.006	0.003	0.003	0.003	0.002	0.002	0.001	0.000	0.001
Fallow		0.155c	0.168a	0.166	0.151	0.060ab	0.060a	0.052	0.072a	0.037a	0.024b	0.023b	0.031ab
Mixture		0.174a	0.176a	0.167	0.156	0.064a	0.064a	0.050	0.071a	0.029b	0.028ab	0.024ab	0.030ab
Red clover		0.165b	0.181a	0.163	0.154	0.062a	0.062a	0.056	0.073a	0.029b	0.025b	0.023b	0.029b
Timothy		0.179a	0.154b	0.164	0.152	0.058b	0.058b	0.049	0.065b	0.030b	0.029a	0.027a	0.034a
SEM		0.003	0.006	0.005	0.005	0.003	0.002	0.003	0.002	0.002	0.002	0.000	0.001
Tests of Between-Subjects effects													
Treatment	DF	Significance level											
Fertilizer	2	ns	ns	ns	ns	ns	ns	ns	*	ns	ns	**	ns
Crop	3	**	**	ns	ns	**	**	ns	**	***	**	**	**
Fert × Crop	6	*	ns	ns	ns	ns	***	ns	ns	ns	ns	ns	*
Tests of Within-Subjects effects													
Treatment	DF	Significance level											
Time	3	***				***				***			

Treatment	DF	*Firmicutes*				*Nitrospirae*				*Rhizobiales*			
		2014		2015		2014		2015		2014		2015	
		June	Sept.	July	Sept.	June	Sept.	July	Sept.	June	Sept.	July	Sept.
Control		0.014	0.009	0.010	0.007	0.006	0.007	0.005	0.008	0.11	0.11	0.102	0.085
Organic		0.012	0.008	0.012	0.008	0.006	0.007	0.004	0.008	0.12	0.11	0.105	0.100
Synthetic		0.011	0.009	0.011	0.006	0.006	0.006	0.004	0.008	0.11	0.12	0.108	0.086
SEM		0.001	0.001	0.002	0.001	0.0005	0.0005	0.0004	0.001	0.005	0.005	0.005	0.005
Fallow		0.012	0.010	0.011	0.009	0.007	0.007	0.005	0.009a	0.10	0.101b	0.101	0.082
Mixture		0.012	0.009	0.014	0.007	0.006	0.007	0.004	0.006b	0.12	0.122a	0.110	0.093
Red clover		0.013	0.008	0.009	0.006	0.006	0.007	0.004	0.008ab	0.12	0.121ab	0.105	0.094
Timothy		0.014	0.008	0.011	0.007	0.005	0.006	0.004	0.007ab	0.12	0.106ab	0.106	0.091
SEM		0.001	0.001	0.002	0.001	0.0005	0.0006	0.0005	0.001	0.005	0.005	0.005	0.005
Tests of Between-Subjects effects													
Treatment	DF	Significance level											
Fertilizer	2	ns	ns	ns	ns	ns	ns	ns	ns	ns	ns	ns	ns
Crop	3	ns	ns	ns	ns	ns	ns	ns	**	ns	*	ns	ns
Fert × Crop	6	ns	ns	ns	ns	ns	ns	ns	ns	ns	ns	ns	ns
Tests of Within-Subjects effects													
Treatment	DF	Significance level											
Time	3		***					***				***	

Sept., September; SEM, standard error of means; Fert, fertilizer treatment; CT, crop treatment; DF, degrees of freedom; ns, not significant; * when $P < 0.05$, ** when $P < 0.01$, *** when $P < 0.001$; Different letters in a column indicate significant differences from fertilizer and crop treatment; Time, sampling time point. The denominator DF of fertilizer, crop, fertilizer and crop interaction, and time are 6, 27, 27, and 80 respectively.

Figures:

A

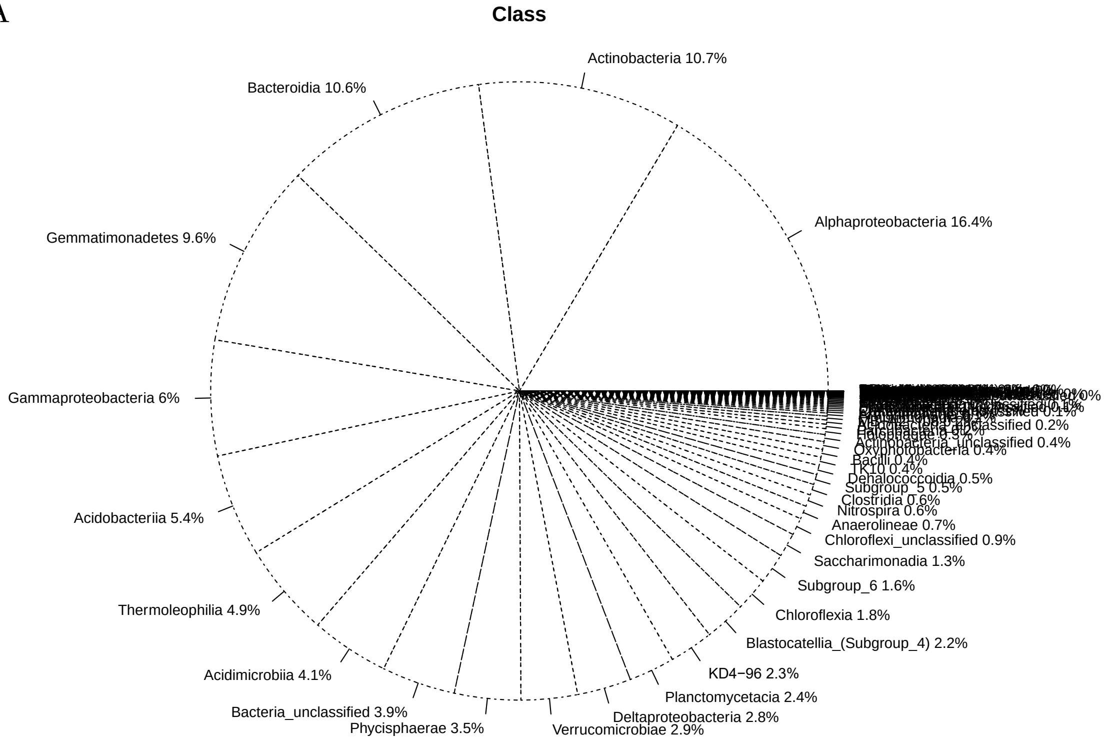
Class
Actinobacteria 10.7%
Bacteroidia 10.6%
Alphaproteobacteria 16.4%
Gemmatimonadetes 9.6%
Gammaproteobacteria 6%
Acidobacteriia 5.4%
Thermoleophilia 4.9%
Acidimicrobiia 4.1%
Bacteria_unclassified 3.9%
Phycisphaerae 3.5%
Verrucomicrobiae 2.9%
Deltaproteobacteria 2.8%
Planctomycetacia 2.4%
KD4−96 2.3%
Blastocatellia_(Subgroup_4) 2.2%
Chloroflexia 1.8%
Subgroup_6 1.6%
Saccharimonadia 1.3%
Chloroflexi_unclassified 0.9%
Anaerolineae 0.7%
Nitrospira 0.6%
Clostridia 0.6%
Subgroup_5 0.5%
Dehalococcoidia 0.5%
TK10 0.4%
Bacilli 0.4%
Oxyphotobacteria 0.4%
Actinobacteria_unclassified 0.4%

B

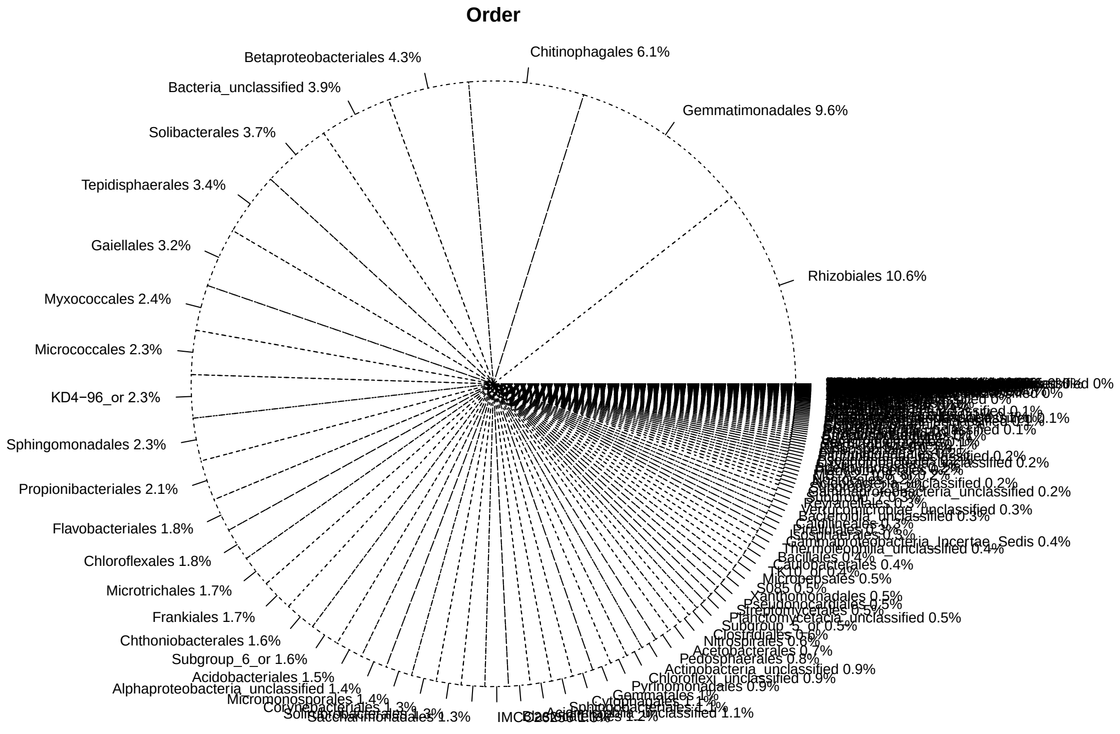
Order
Chitinophagales 6.1%
Betaproteobacteriales 4.3%
Bacteria_unclassified 3.9%
Gemmatimonadales 9.6%
Solibacterales 3.7%
Tepidisphaerales 3.4%
Gaiellales 3.2%
Rhizobiales 10.6%
Myxococcales 2.4%
Micrococcales 2.3%
KD4−96_or 2.3%
Sphingomonadales 2.3%
Propionibacteriales 2.1%
Flavobacteriales 1.8%
Chloroflexales 1.8%
Microtrichales 1.7%
Frankiales 1.7%
Chthoniobacterales 1.6%
Subgroup_6_or 1.6%
Acidobacteriales 1.5%
Alphaproteobacteria_unclassified 1.4%
Micromonosporales 1.4%
Saccharimonadales 1.3%
Gammaproteobacteria_Incertae_Sedis 0.4%
Thermoleophilia_unclassified 0.4%
Caulobacterales 0.4%
Bacillales 0.4%
Microbacteriales 0.5%
S085 0.5%
Xanthomonadales 0.5%
Pseudonocardiales 0.5%
Streptomycetales 0.5%
Planctomycetacia_unclassified 0.5%
Subgroup_5_or 0.5%
Clostridiales 0.6%
Nitrospirales 0.6%
Acetobacterales 0.7%
Pedosphaerales 0.8%
Actinobacteria_unclassified 0.9%
Chloroflexi_unclassified 0.9%
Pyrinomonadales 0.9%
Gemmatales 1%
Gammaproteobacteria_unclassified 0.2%
Bacteroidia_unclassified 0.3%

C

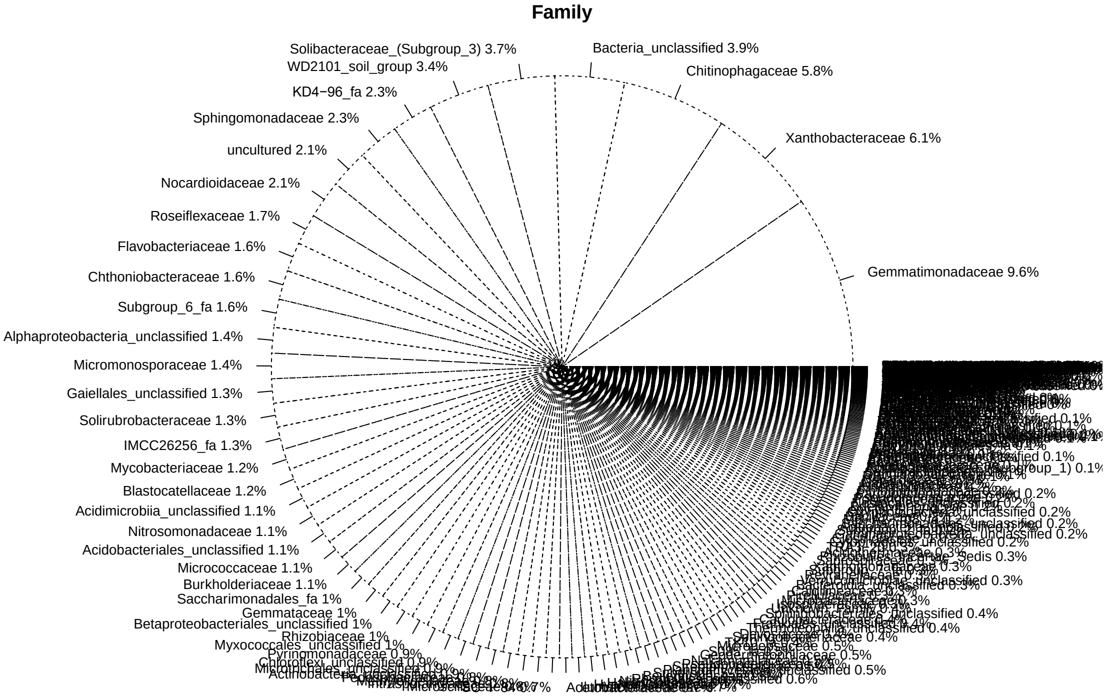

D

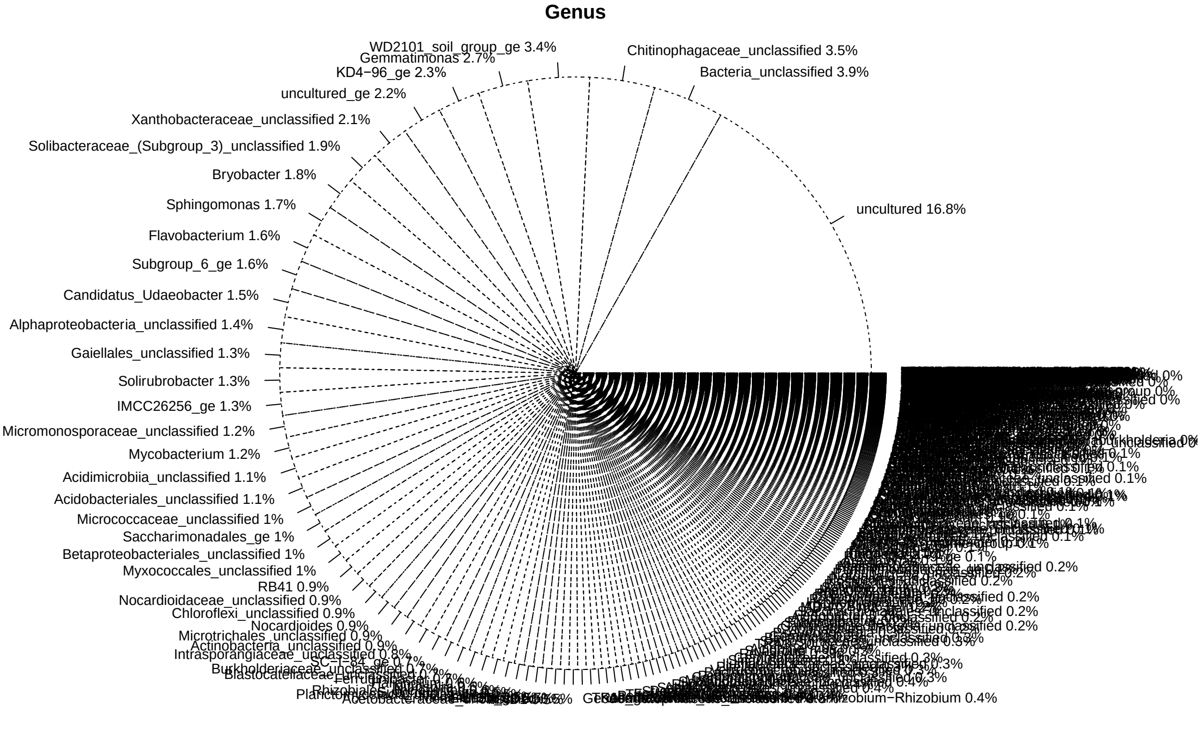

Figure S1 Soil bacterial taxonomy information at Class (A), Order (B), Family (C), and Genus (D) level.

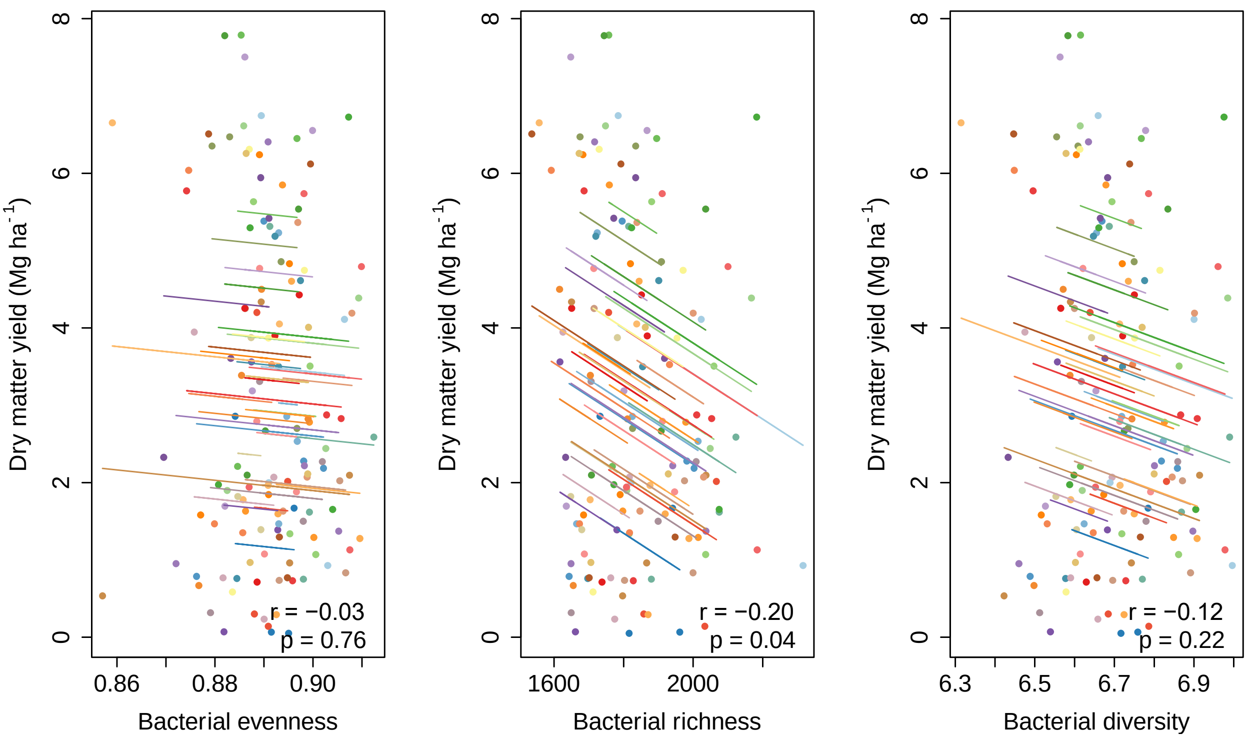

Figure S2 Overall repeated measures correlation of bacterial community diversity measures to crop dry matter yield.

A

B

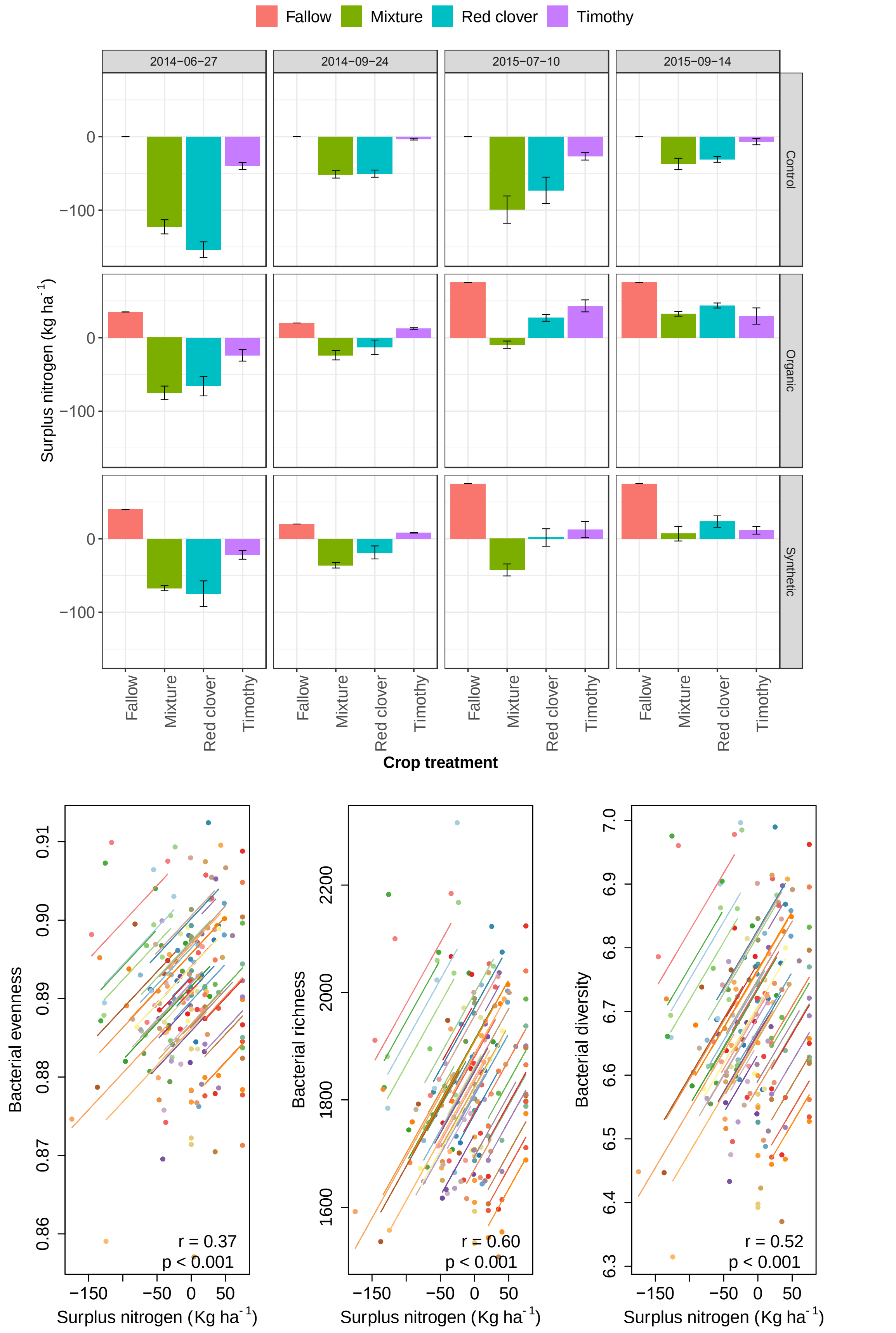

Figure S3 Dynamic change of surplus nitrogen (kg N ha^{-1}) (Fertilization N – Crop N yield) under

fertilizer and crop treatments (A) and overall repeated measures correlations of surplus nitrogen (Fertilization N – Crop N yield) to bacterial community diversity measures (B).

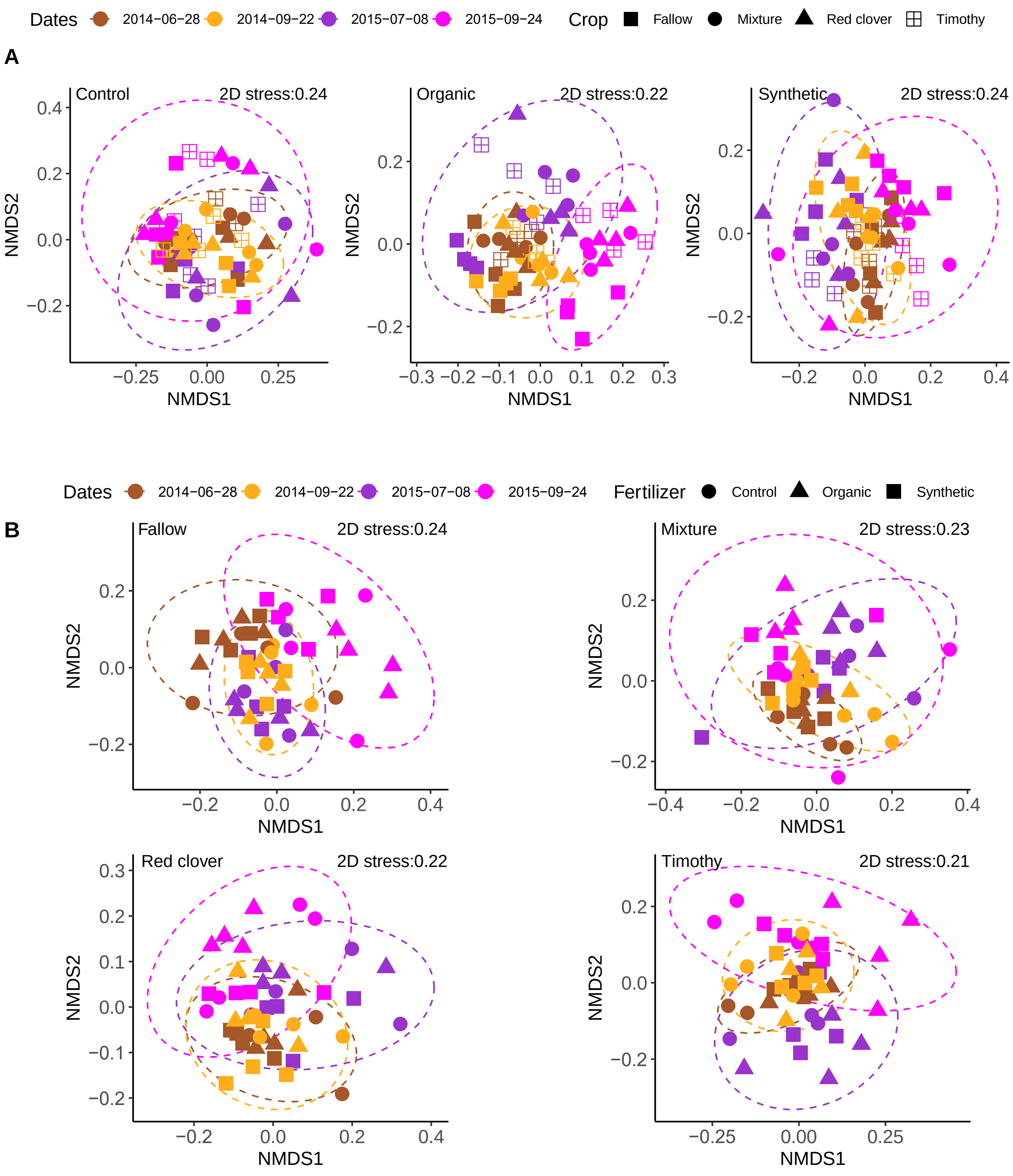

Figure S4 Nonmetric multidimensional scaling (NMDS) of soil bacterial community composition within fertilizer (A) and crop (B) treatments. Fallow: no plant cover; mixture, red clover – timothy mixture; Control: no fertilization; organic: organic fertilizer; synthetic: synthetic fertilizer.

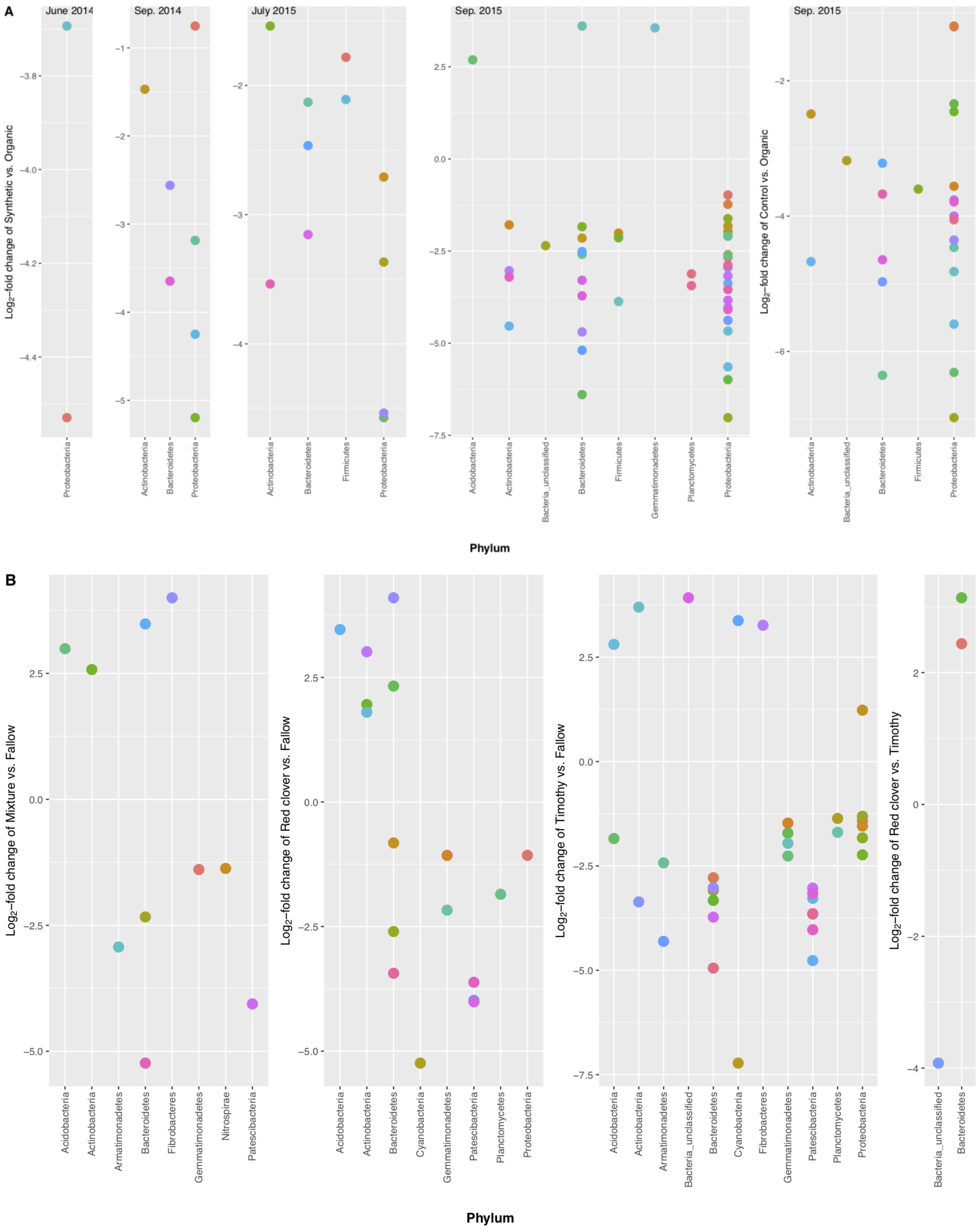

Figure S5 Log_2fold change of differential OTUs in synthetic vs. organic fertilizer treatment over time and control vs. organic in September 2015 (A), and planted plots vs. fallow and red clover vs. timothy at the end of the experiment (B). Each coloured circle represents an OTU. Control: no fertilization; organic: organic fertilizer; synthetic: synthetic fertilizer. Fallow: no plant cover; mixture, red clover – timothy mixture.

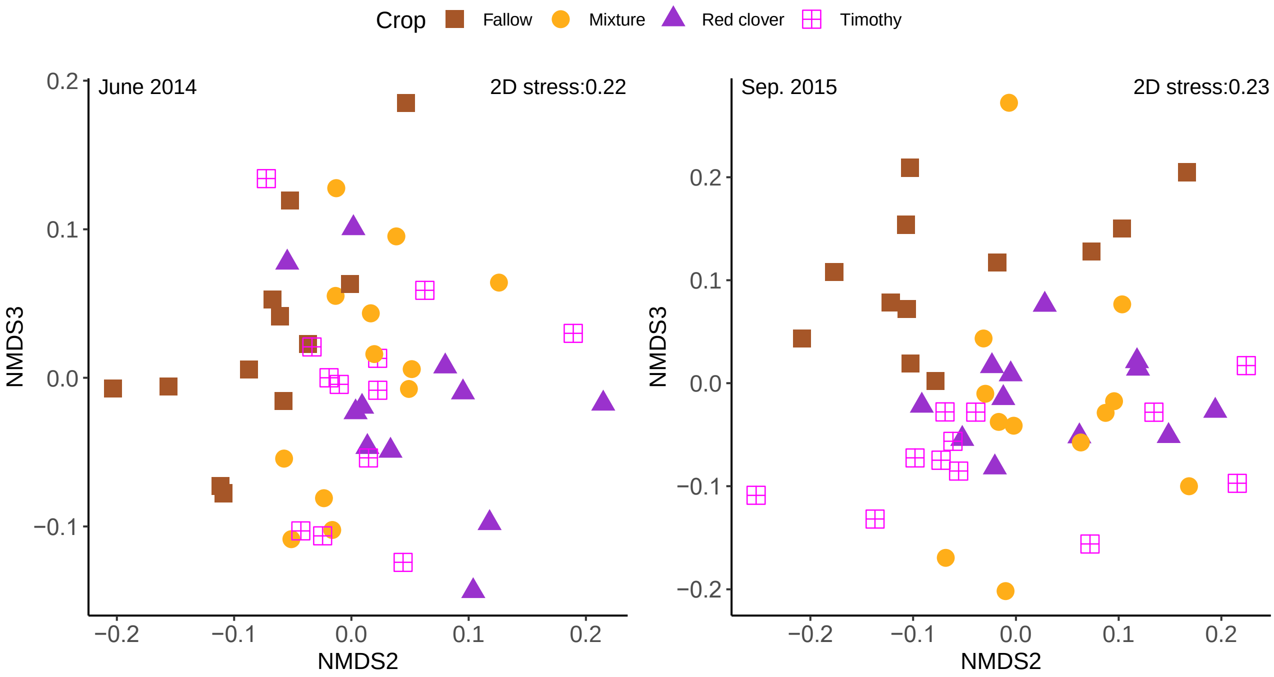

Figure S6 Nonmetric multidimensional scaling (NMDS) of soil bacterial community composition under different crop treatments in terms of NMDS2 to NMDS3 in June 2014 and September 2015. Fallow: no plant cover; mixture, red clover – timothy mixture;

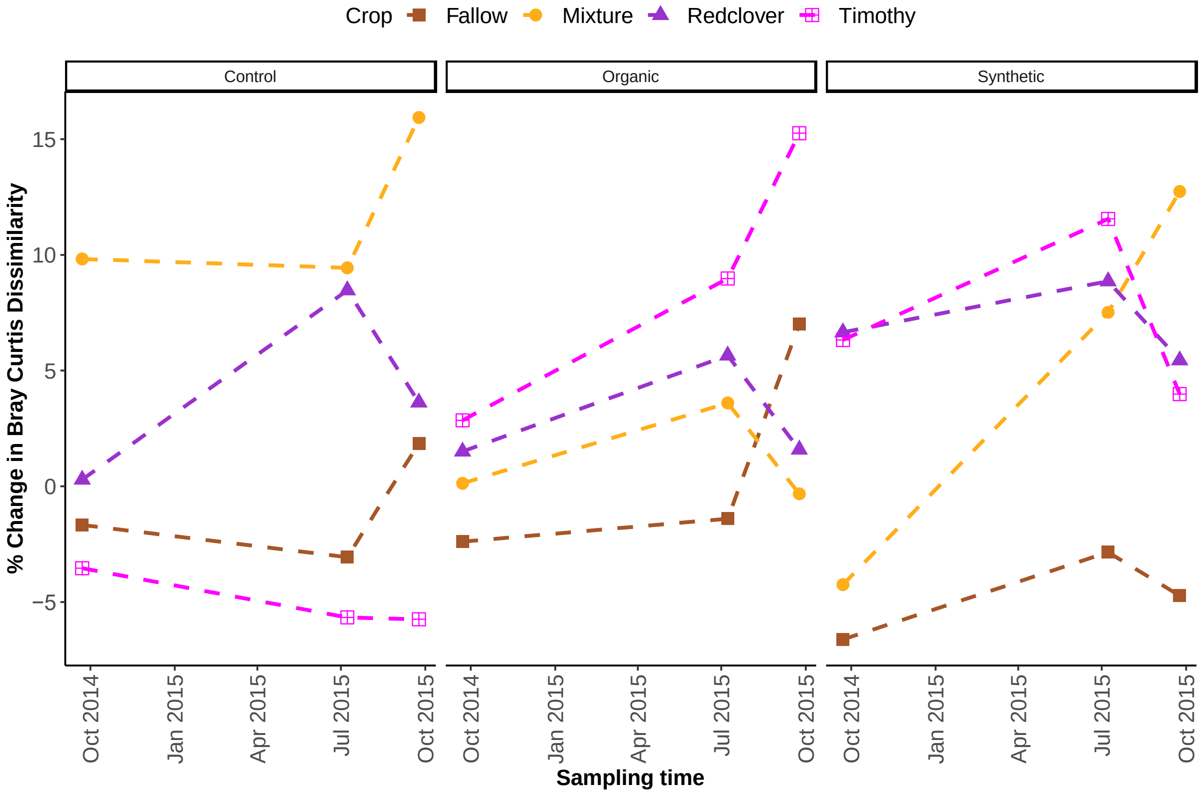

Figure S7 Temporal changes of bacterial composition in Bray-Curtis dissimilarity. The averaged pairwise dissimilarities from June 2014 were calculated and presented as differences in percentage. Fallow: no plant cover; mixture, red clover – timothy mixture; Control: no fertilization; organic: organic fertilizer; synthetic: synthetic fertilizer.
